# Effect of Transition Metal Doping on the Structural, Morphological, and Magnetic Properties of NiFe_2_O_4_

**DOI:** 10.3390/ma15092996

**Published:** 2022-04-20

**Authors:** Thomas Dippong, Oana Cadar, Erika Andrea Levei

**Affiliations:** 1 Faculty of Science, Technical University of Cluj-Napoca, 76 Victoriei Street, 430122 Baia Mare, Romania; dippong.thomas@yahoo.ro; 2 INCDO-INOE 2000, Research Institute for Analytical Instrumentation, 67 Donath Street, 400293 Cluj-Napoca, Romania; oana.cadar@icia.ro

**Keywords:** nickel ferrite, nanoparticle, divalent metal doping, magnetic properties

## Abstract

Sol-gel route followed by thermal treatment was used to produce NiFe_2_O_4_ doped with transition metal ions (Zn^2+^, Mn^2+^, Co^2+^). The structural, morphological, and magnetic properties of the doped NiFe_2_O_4_ were compared with those of virgin NiFe_2_O_4_. The metal-glyoxylates’ formation and decomposition as well as the thermal stability of the doped and virgin ferrites were assessed by thermal analysis. The functional groups identified by Fourier-transform infrared spectroscopy confirmed the decomposition of metal nitrates, the formation and decomposition of precursors, and the formation of the SiO_2_ matrix. The X-ray diffraction indicated that the sol-gel synthesis produced single-phase crystalline ferrites in case of virgin, Zn^2+^ and Co^2+^-doped Ni-ferrites. By doping with Mn^2+^, several secondary phases derived from the SiO_2_ matrix accompanied the crystalline spinel ferrite. The crystallite sizes depended on the annealing temperature and type of doping ion. The gradual increase of lattice parameters suggested the uniform distribution of doping metal ions in the NiFe_2_O_4_ lattice. The saturation magnetization, remanent magnetizations, coercivity, and anisotropy were found to depend on the doping ion, annealing temperature, and particle size. The high saturation magnetization values of the obtained nanocomposites make them suitable for a wide range of applications in the field of sensors development and construction.

## 1. Introduction

Spinel ferrites are the topic of numerous studies due to their magnetic nature and crystalline structure. Small changes of the particle size, composition or presence of surface effects give them unique magnetic features [1]. Nanosized spinel ferrites received a huge amount of interest due to their low cost, excellent chemical stability, moderate saturation magnetization, high surface area, high wear resistance, low density, low thermal expansion coefficient, and low toxicity to both human health and environment [2,3,4]. These ferrites are promising candidates for a broad range of applications in the industry (magnetic recording media, photoelectric devices, sensors, magnetic pigments, photocatalysts in dye degradation, controlled signal transformation, storage devices, batteries, solar cells) and biomedicine (controlled drug delivery, tumor treatment, magnetic resonance imaging, bio-magnetic separation, cellular therapy, tissue repair, cell separation, and biosensing) [1,2,3,4,5,6,7,8].

The NiFe_2_O_4_ is a soft magnetic, semiconducting material with ferromagnetic properties, prominent electrical resistivity, low conductivity, low eddy current loss, high chemical stability, catalytic behavior, etc. [1,5,9,10]. NiFe_2_O_4_ possesses an inverse spinel structure, with the Fe^3+^ ions distributed equally between the tetrahedral (A) and octahedral (B) sites while, the Ni^2+^ ions occupy the octahedral (B) sites [1,5,11]. 

The ferrite structure and properties are sensitive to the synthesis method, additive substitution, and calcination process [12,13,14]. Doping with transition metal ions, such as Co^2+^, Ni^2+^, or Zn^2+^, is an effective way to improve and control the structure and consequently the optical, electrical, dielectric, and magnetic properties of nanosized NiFe_2_O_4_ [1,7,8,15,16,17]. The doping with transition metal ions into spinel ferrite structure changes the cations’ distribution between the tetrahedral (A) and octahedral (B) sites, leading to different magnetic properties. The dopant ion may also change the energy of the grain boundaries, acting as a driving force of the grain growth [1]. The electrical resistivity can also be improved by doping the host matrices with smaller divalent cations or by controlling their microstructures [8]. Zn^2+^ doping disturbs the cation distribution, enhances the dielectric and magnetic properties [18]. The substitution of NiFe_2_O_4_ with magnetic divalent transition metal ions like Mn^2+^ led to appealing magnetic and electrical features [2,12,14,19,20]. By adjusting the Mn-to-Ni ratio in the ferrite, the magnetic properties of the ferrite can be controlled [2]. The Ni^2+^ ions’ addition overcomes the grain formation, leading to low surface roughness [13,21].

The physico-chemical properties of nanosized ferrites are highly influenced by the synthesis route, dopant ion nature and amount, as well as the presence of structural order–disorder effects [1,5]. The annealing temperature influences the grain boundary migration and grain boundary diffusion, which further determines the grain shape, grain size, core density, and microstructure [4]. The synthesis route is a key factor to obtain high-purity nanoferrites [1]. Several methods for producing nanoferrites, such as sol-gel, co-precipitation, refluxing, hydrothermal, mechano-chemical, solid-state, precursor, auto combustion, microwave plasma, microemulsion, mechanical alloying, etc. are described in the literature [1,5,7]. Among these, to produce ferrite nanocomposites, the sol-gel method is one of the most-used approaches due to its simplicity, low cost, low processing temperature, and good control over the structure, physico-chemical properties, surface properties, and magnetic behavior [22]. To obtain spinel ferrites by the sol-gel method, nitrate salts are frequently used, as they act as water-soluble, low-temperature oxidizing agents [23]. Solvothermal synthesis allows the large-scale production of ferrites with controlled size and shape by choosing the appropriate aqueous or non-aqueous solvent mixture, by varying the synthesis temperature, pressure, and reaction time [3]. The microwave-assisted synthesis of ferrites has a lower yield than hydrothermal or thermal-decomposition methods [3]. The co-precipitation method is another frequently used method to produce nanoparticles with a specific shape and size [22]. The major disadvantage associated with the ferrite production by co-precipitation is the poor crystallinity of the resulting NPs, that may be enhanced by subsequent heat treatment [3]. Auto-combustion is a simple and low-cost process that requires a short reaction time and low energy consumption [4,24]. The ferrites prepared by this method have homogeneous chemical composition, high-purity, and good sinterability [24]. In the modified sol-gel method, the reactants are mixed with tetraethyl orthosilicate (TEOS), the sol is exposed to air until the gelation of the silica (SiO_2_) network, the gels are thermally treated to obtain carboxylate precursors that are further thermally decomposed into the oxidic systems. This method is versatile, simple, and effective in producing pure nanoparticles, but has the drawback of having the presence of amorphous phases at low annealing temperatures and of secondary crystalline phases at high annealing temperatures [25]. Among different coating materials, mesoporous SiO_2_ is non-toxic and biocompatible, allows the control of the particle growth, minimizes the nanoparticles agglomeration, improves their stability, enhances the magnetic guidability and bio-compatibility, and favors the conjugation with functional groups, [26,27,28,29].

The paper aims to investigate the structural, morphological and magnetic properties of virgin NiFe_2_O_4_ and NiFe_2_O_4_ doped with transition divalent metal ions Zn^2+^ (Zn_0.15_Ni_0.85_Fe_2_O_4_), Mn^2+^ (Mn_0.15_Ni_0.85_Fe_2_O_4_), and Co^2+^ (Co_0.15_Ni_0.85_Fe_2_O_4_) embedded in a SiO_2_ matrix produced by sol-gel route, followed by thermal treatment at various temperatures. This study is of particular interest due to the lack of information on the effect of dopant nature (Zn^2+^, Mn^2+^ and Co^2+^) on the size and magnetic properties of mixed M_0.15_Ni_0.85_Fe_2_O_4_ (M=Co, Mn and Zn) type ferrites embedded in SiO_2_ matrix. Because the oxidic phases at low temperatures are poorly crystalline or even amorphous, the desired surface properties and crystallinity can be achieved by using specific annealing conditions. Besides, the reactivity of the amorphous phases allows their participation in a variety of chemical transformations. In this regard, the X-ray diffraction (XRD) parameters were compared for different annealing temperatures to get important structural information. The thermal (TG-DTA) analysis and Fourier transform infrared (FT-IR) spectroscopy depicted the formation and decomposition of metallic glyoxylate precursors, the stability of the produced ferrites and formation SiO_2_ matrix. A special emphasis was given to the evolution of magnetic properties (saturation magnetization (*M_S_*), remanent magnetization (*M_R_*), coercivity (*H_C_*), and anisotropy (*K*)) with the increase of annealing temperature and the type of doping ion. 

## 2. Materials and Methods

### 2.1. Reagents

Iron (III) nitrate nonahydrate (Fe(NO_3_)_3_∙9H_2_O, 98%), nickel (II) nitrate hexahydrate (Ni(NO_3_)_2_∙6H_2_O, 99%), zinc (II) nitrate hexahydrate (Zn(NO_3_)_2_∙6H_2_O, 98%), manganese (II) nitrate tetrahydrate (Mn(NO_3_)_2_∙4H_2_O, 98%), cobalt (II) nitrate hexahydrate (Co(NO_3_)_2_∙6H_2_O, 98%), 1,2 ethanediol (1,2-ED, 99%), tetraethyl orthosilicate (TEOS, 99%) and ethanol 96% (Merck, Darmstadt, Germany) were used in the synthesis. 

### 2.2. Synthesis

**Figure 1:**
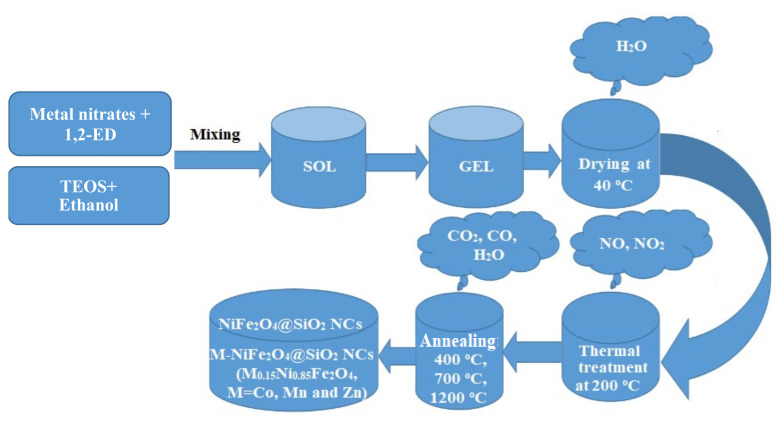
Schematic diagram of virgin and doped NiFe_2_O_4_ (Zn-NiFe_2_O_4_, Mn-NiFe_2_O_4_, Co-NiFe_2_O_4_) synthesis.

NiFe_2_O_4_ and M-NiFe_2_O_4_ embedded in SiO_2_ (M_0.15_Ni_0.85_Fe_2_O_4_, M=Co, Mn and Zn) nanocomposites, containing 70 wt.% ferrite and 30 wt.% SiO_2_, were prepared by modified sol-gel method by using a M/Ni/Fe molar ratio of 0.15/0.85/2. A schematic diagram of the synthesis method is given in Figure 1. To prepare the sols, the metal nitrates were mixed with 1,2-ED, TEOS and ethanol by using a NO_3_^−^/ED/TEOS molar ratio of 1/1/0.50. The resulting sols were stirred continuously for 30 min and maintained in open air, at room temperature until gelation occurs. The formed gel embedded a homogenous mixture of metal nitrates and 1,2-ED. As the production of high-purity gels with high crystallites size is favored by a thermal pretreatment before annealing [5], the obtained gels were grinded, dried at 40 and 200 °C, and annealed at 400 °C (5 h), 700 °C (5 h) and 1000 °C (5 h), respectively, by using a LT9 muffle furnace (Nabertherm, Lilienthal, Germany).

By heating the gels at 200 °C the redox reactions between the nitrates and 1,2-ED take place in the pores of the SiO_2_ matrix resulting a mixture of Fe(III), Ni(II), and M(II) glyoxylates. The mixtures of glyoxylates around 300 °C decompose into metal oxides that reacts at temperatures above 300 °C and forms the ferrites. SiO_2_ has de role of a spacer between the nanoparticles, reducing the particle agglomeration [14,29]. 

### 2.3. Characterization

The thermal behavior was investigated by thermogravimetric (TG) and differential thermal analysis (DTA) by using a Q600 SDT (TA Instruments, Newcastle, DE, USA) thermal analyzer, in air up to 1000 °C, at 5 °C/min. The FT-IR spectra were recorded by using a Spectrum BX II (Perkin Elmer, Waltham, MA, USA) Fourier-transform infrared spectrometer on pellets containing 1% (*w*/*w*) sample in KBr. The X-ray diffraction patterns were recorded by using a D8 Advance (Bruker, Karlsruhe, Germany) diffractometer, operating at room temperature, 40 kV, and 40 mA with CuKα radiation (λ = 1.54060 Å). The Co/Ni/Fe (Co_0.15_Ni_0.85_Fe_2_O_4_@SiO_2_), Mn/Ni/Fe (Mn_0.15_Ni_0.85_Fe_2_O_4_@SiO_2_), and Zn/Ni/Fe (Zn _0.15_Ni_0.85_Fe_2_O_4_@SiO_2_) molar ratios were verified by inductively coupled plasma optical emission spectrometry (ICP-OES) by using a Perkin Elmer Optima 5300 DV (Norwalk, CT, USA) spectrometer, after microwave digestion with aqua regia. The nanoparticles morphology was studied by transmission electron microscopy (TEM) and scanning electron microscopy (SEM) on samples deposited from suspension onto carbon-coated copper grids by using an HD-2700 (Hitachi, Tokyo, Japan) transmission electron microscope and a SU8230 (Hitachi, Tokyo, Japan) scanning electron microscope. A cryogen-free vibrating-sample magnetometer (Cryogenic Limited, London, UK) was used for the magnetic measurements.

## 3. Results and Discussion

### 3.1. Thermal Analysis

**Figure 2:**
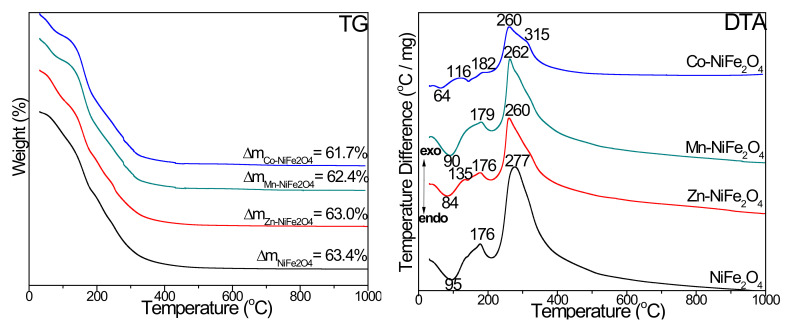
TG and DTA curves of virgin and doped NiFe_2_O_4_ (Zn-NiFe_2_O_4_, Mn-NiFe_2_O_4_, Co-NiFe_2_O_4_) dried at 40 °C.

The TG/DTA curves of virgin and doped NiFe_2_O_4_ samples dried at 40 °C are presented in Figure 2. The DTA curve shows three processes: (I) loss of moisture and physically adsorbed water suggested by the endothermic effects at 64–95 °C, (II) formation of metal-glyoxylate precursors shown by the exothermic effects at 116–182 °C and (III) decomposition of glyoxylate precursors into ferrites as indicated by the exothermic effect at 260–315 °C. 

In case of virgin NiFe_2_O_4_ and Mn-doped NiFe_2_O_4_, both the glyoxylate precursors formation and decomposition into NiFe_2_O_4_ and Mn_0.15_Ni_0.85_Fe_2_O_4_ take place in single stages. The total mass loss is 63.4% for NiFe_2_O_4_ and 62.4% for Mn_0.15_Ni_0.85_Fe_2_O_4_, respectively. Zn-doped NiFe_2_O_4_ shows the same three processes, but the formation of metal-glyoxylate precursors takes place in two stages: the Ni- and Zn-glyoxylates are formed at 135 °C, whereas the Fe- glyoxylate at 176 °C. The total mass loss shown on the TG curve is 63%. In case of Co-doped NiFe_2_O_4_, both the formation and the decomposition of the glyoxylate precursors occur in two stages: Co- and Ni- glyoxylates are formed at 166 °C and decomposed at 260 °C, whereas Fe- glyoxylate is formed at 182 °C and decomposed at 315 °C. During the metal glyoxylates decomposition, the resulted Fe_2_O_3_ reacts with Co_3_O_4_ and NiO to form Co_0.15_Ni_0.85_Fe_2_O_4_ [7,8]. The TG curve indicate a total mass loss of 61.7%. Thus, between 260 and 277 °C, the virgin Ni-ferrite, as well as the Zn- and Mn-doped ferrites are formed, whereas the Co-doped ferrite is formed at 315 °C. The mass losses are comparable, the highest mass loss being recorded for the virgin Ni-ferrite and the lowest mass loss for Co doped Ni-ferrite.

### 3.2. Fourier-Transform Infrared Spectroscopy

**Figure 3:**
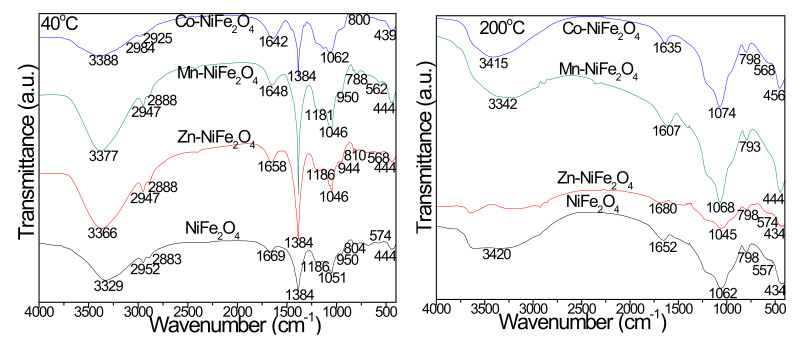
FT-IR spectra of virgin and doped NiFe_2_O_4_ (Zn-NiFe_2_O_4_, Mn-NiFe_2_O_4_, Co-NiFe_2_O_4_) heated at 40 and 200 °C.

The FT-IR spectra offers data on the presence of different functional groups, molecular geometry and inter-molecular interactions [1]. In samples heated at 40 °C, the FT-IR spectra (Figure 3) display an intense band at 1384 cm^−1^ specific to nitrate groups [25,30], which disappears for samples heated at 200 °C, indicating the nitrates decomposition.

The bands at 2984–2952 and 2888–2925 cm^−1^ are attributed to C-H bond-asymmetric and symmetric stretching in 1,2ED. The band at 1669–1642 cm^−1^ is assigned to the O-H stretching and bending in both 1,2ED and adsorbed water. The band at 3388–3329 cm^−1^ is assigned to the O-H stretching and intermolecular hydrogen bonds in 1,2ED at 40 °C, and the band at 950–944 cm^−1^ is assigned to -OH stretching and Si-OH deformation vibration following the hydrolysis of -Si (OCH_2_CH_3_)_4_ groups in TEOS [25,30]. In samples heated to 200 °C, the vibration of C=O in COO^−^ groups indicated by the bands at 1680–1607 cm^−1^, confirms the coordination of carboxylate groups by metal ions and the formation of a chelated complex [25,30]. The band at 574–557 cm^−1^ is assigned to tetrahedral M-O bonds and cyclic Si-O-Si structures vibrations, whereas the band at 456–434 cm^−1^ is assigned to the octahedral M-O and Si-O bonds vibration [25,30]. The formation of the SiO_2_ matrix is confirmed by the Si-O bond vibration at 439–456 cm^−1^, Si-O-Si cyclic structures vibration at 574–557 cm^−1^, Si-O-Si chains symmetric stretching and bending at 788–810 cm^−1^, and Si-O-Si bonds stretching vibration at 1046–1074 cm^−1^ with shoulder at 1181–1186 cm^−1^ [25,30].

**Figure 4:**
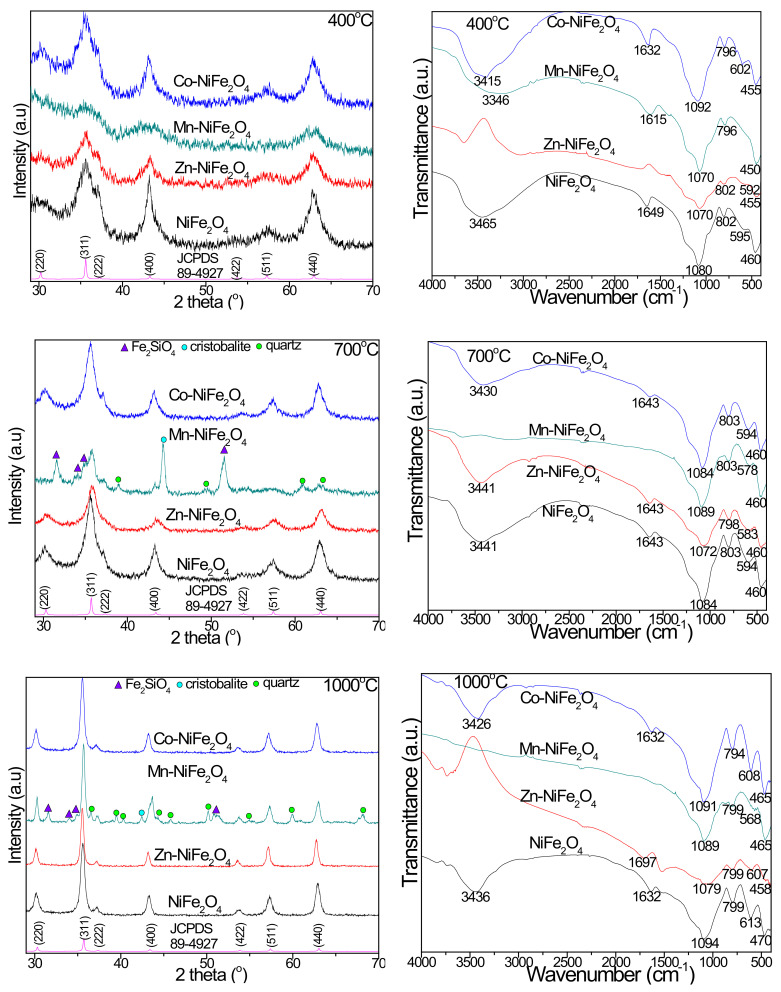
XRD patterns and FT-IR spectra of virgin and doped NiFe_2_O_4_ (Zn-NiFe_2_O_4_, Mn-NiFe_2_O_4_, Co-NiFe_2_O_4_) samples annealed at 400, 700, and 1000 °C.

The FT-IR spectra of virgin and doped NiFe_2_O_4_ (Zn-NiFe_2_O_4_, Mn-NiFe_2_O_4_, Co-NiFe_2_O_4_) annealed at high temperatures (Figure 4) show the presence of the characteristic bands for SiO_2_ matrix: O-H bonds vibration in Si-OH group (3465–3346 cm^−1^), H-O-H bending (1697–1615 cm^−1^), Si-O-Si stretching (1094–1070 cm^−1^), Si-O chains symmetric stretching and bending in the SiO_4_ tetrahedron (803–794 cm^−1^), Si-O bonds vibration (47–450 cm^−1^) and Si-O-Si cyclic structures vibration (613–568 cm^−1^) [5,6,10,11,15]. The absorption band at 470–450 cm^−1^ can be attributed also to the M-O stretching vibration at the octahedral (B) site, whereas that at 613–568 cm^−1^ to the M-O stretching vibration at the tetrahedral site [1,6,25,30], indicating the formation of ferrites with cubic structure [1]. The doping of NiFe_2_O_4_ with larger size and higher atomic weight divalent ions forces the migration of Fe^3+^ ions to the octahedral (B) sites leading to a decrease of the tetrahedral vibration frequency and an increase of the octahedral vibration frequency [9]. 

### 3.3. X-ray Diffraction

The XRD patterns of virgin and doped NiFe_2_O_4_ annealed at 400, 700, and 1000 °C are presented in Figure 4. The samples annealed at 400 °C display the diffraction peaks corresponding to the reflection planes of (220), (311), (222), (400), (422), (511), and (440), confirming the presence of low-crystallized single phase NiFe_2_O_4_ (JCPDS card no 89-4927) [31]), with no detectable impurity phases [1,25]. By increasing the annealing temperature (700 and 1000 °C), in case of NiFe_2_O_4_, Zn-(Zn-NiFe_2_O_4_) and Co-dopped (Co-NiFe_2_O_4_) NiFe_2_O_4_ single phase ferrites are obtained. The increase of the diffraction lines’ intensity indicates the increase of crystallinity and particle size [5]. In case of Mn-dopped NiFe_2_O_4_ (Mn-NiFe_2_O_4_), both at 700 and 1000 °C, cristobalite (JCPDS card no. 89-3434 [31], quartz (JCPDS card 85-0457 [31]) and Fe_2_SiO_4_ (JCPDS card no.87-0315 [31]) are also identified as secondary phases. The presence of secondary phases could be explained by the higher mobility of cations and strain variation induced by the annealing process, that also slightly shifts the 2θ positions and broadens the peaks, concomitantly with the increase of crystallite sizes [31]. The formation of Fe_2_SiO_4_ could be attributed to the reducing conditions generated during the carboxylate precursors decomposition that partially reduce the Fe^3+^ ions to Fe^2+^ ions within the SiO_2_ matrix pores, which further reacts with SiO_2_ to form Fe_2_SiO_4_ [12,13,32].

The variation of the oxygen atoms’ positions results in structural distortion of the FeO_6_, FeO_4_, and NiO_6_ complexes that highly disturb the NiFe_2_O_4_ lattice, leading to structural changes with high impact on the physico-chemical properties [5]. In case of doping with Mn^2+^ ions, the diffraction peak situated near 2θ = 35° are slightly shifted. Some possible explanations could be the Mn^2+^ ions that enter in the octahedral (B) sites as well as the larger radius of Mn^2+^ (0.80 Å) than of Ni^2+^ (0.72 Å) [15]. The crystallite size (D) calculated from the most intense diffraction peaks (311), lattice constant (a), unit cell volume (V), bulk density (d_p_), X-ray density (d_XRD_), porosity (P), and hopping length in tetrahedral (L_A_) and octahedral (L_B_) sites [6,8,33,34,35] are shown in Table 1. XRD parameters are influenced not only by the crystallite size, lattice strain and defects, but also by the annealing temperature and doping ions [6]. The sharpening and narrowing of the diffraction peaks suggest the crystallite size become more obvious with the annealing temperature [16]. At high annealing temperatures (1000 °C), a significant agglomeration takes place without subsequent recrystallization, supporting the formation of a single crystal instead of a polycrystal structure [5,36]. 

The lattice constant (a) increases, whereas the X-ray density (d_XRD_) decreases with increasing crystallite size. Some possible explanations could be the surface tension decrease caused by the size effect and the expansion of unit cell by replacing Ni^2+^ with Zn^2+^, Co^2+^, and Mn^2+^ ions [6,17,33]. Considering the small difference between the atomic weight of Ni^2+^ and Mn^2+^ ions, the d_XRD_ variation may be attributed to the changes of the lattice constant (a) [37]. The lattice constant (a) shows a linear behavior and it follows Vegard’s law. The differences between the lattice parameter of investigated samples were attributed to the different ionic radii of Fe^3+^ (tetra: 0.49; octa: 0.64 Å), Zn^2+^ (tetra: 0.60; octa: 0.74 Å), Ni^2+^ (tetra: 0.54; octa: 0.78 Å), Mn^2+^ (tetra: 0.58; octa: 0.69 Å), and Co^2+^ (tetra: 0.58; octa: 0.74 Å) [17,33,35]. The decrease of porosity (P) with the increase of annealing temperature may be a consequence of the rapid densification during the annealing process [6,17,33].

### 3.4. Chemical Analysis

The M/Ni/Fe molar ratio calculated based on Co, Mn, Zn, Ni and Fe concentrations measured by ICP-OES confirmed the theoretical elemental composition of the obtained NCs (Table 1). In all cases, the best fit of experimental and theoretical data was remarked for samples annealed at 1000 °C. In case of Mn-dopped NiFe_2_O_4_ annealed at 700 and 1000 °C, the Mn/Ni/Fe molar ratio could not be calculated based on the metal concentrations, due to the presence of Fe_2_SiO_4_ as secondary phase.

### 3.5. Transmission and Scanning Electron Microscopy

**Figure 5:**
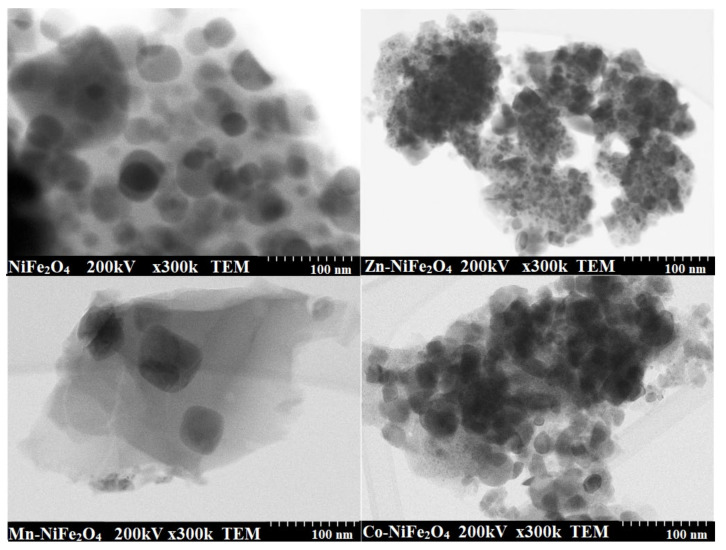
TEM images of virgin and doped NiFe_2_O_4_ (Zn-NiFe_2_O_4_, Mn-NiFe_2_O_4_, Co-NiFe_2_O_4_) NCs annealed at 1000 °C.

The TEM images (Figure 5) reveal irregularly shaped particles that form agglomerates. As a result of the doping with Zn^2+^ and Co^2+^ ions, a decrease of the particle size from 29 nm (NiFe_2_O_4_) to 10 nm (Zn-NiFe_2_O_4_) and 21 nm (Co-NiFe_2_O_4_) was observed, whereas by doping with Mn^2+^ ion, the particle size increases to 43 nm (Mn-NiFe_2_O_4_). 

The variation of particle size by doping may be determined by the different kinetics of metal oxides’ formation reaction, the different particle growth rate or the presence of structural disorder and strain in the lattice due to different ionic radii [14,37]. The different particle arrangement could be attributed to the formation of well-delimited grains that form solid boundaries. 

The particle agglomeration is frequently observed in case of NCs synthesized by chemical routes and is caused most probably by the assembling tendency of small particles, magnetic nature, and weak surface interaction due to Van der Waals forces [8,9,25,33]. The internal heat energy produced during the annealing may also lead to the agglomeration of particles due to interfacial surface tensions [8,25]. 

The differences obtained between particle and crystallite size result most probably due to the interference of amorphous matrix and of large-size nanoparticles that highly influence the diffraction patterns, by the large fraction of the total number of atoms contained [8]. The crystal-growth rate increase could be attributed to volume expansion and supersaturation reduction of the system at high annealing temperatures, which further leads to increase of the amorphous Fe oxides solubility and crystallization of M_0.15_Ni_0.85_Fe_2_O_4_ when Mn, Zn, and Co diffuse into the crystal structure of NiFe_2_O_4_. When the nucleation rate exceeds the growth rate, small and homogenously distributed particles are obtained. At high annealing temperatures, these particles may join together due to coalescence, formation of crystalline clusters, and joint cementation [8,14,25,37]. 

**Figure 6:**
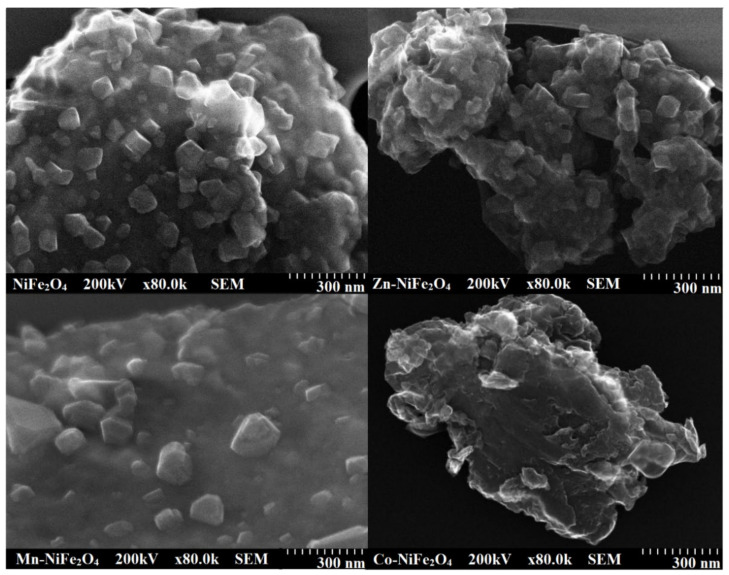
SEM images of virgin and doped NiFe_2_O_4_ (Zn-NiFe_2_O_4_, Mn-NiFe_2_O_4_, Co-NiFe_2_O_4_) NCs annealed at 1000 °C.

The SEM images (Figure 6) indicates agglomerations of homogenous, clearly delimited particles typical of ferrite materials containing magnetic elements [25]. The particles in Zn and Co dopped NiFe_2_O_4_ have a homogenous microstructure with closely packed, irregularly shaped small particles, whereas those in Mn dopped NiFe_2_O_4_ are bigger and more loosely packed. 

### 3.6. Magnetic Properties

**Figure 7:**
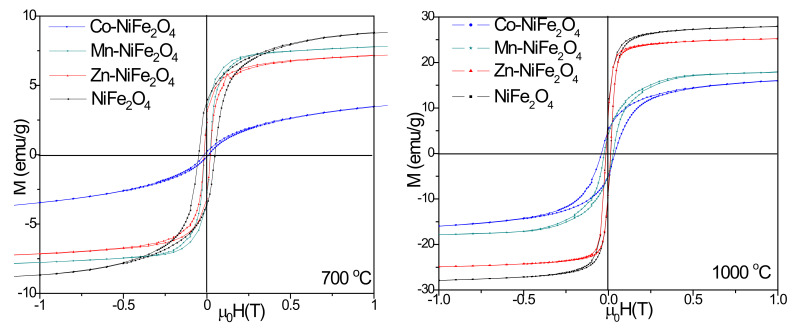
Magnetic hysteresis loops of virgin and doped NiFe_2_O_4_ (Zn-NiFe_2_O_4_, Mn-NiFe_2_O_4_, Co-NiFe_2_O_4_) NCs annealed at 700 and 1000 °C.

All samples display superparamagnetic behavior with well-defined hysteresis loops (Figure 7), but important differences in the magnetic parameters are induced by the doping ions. Small particles contain fewer domain walls and require higher demagnetization force, whereas large particles have a higher probability of domain formation [9].

The saturation magnetization (*M_S_*), remanent magnetization (*M_R_*), coercivity (*H_C_*), squareness (*Sq*), and anisotropy (*K*) of NCs annealed at 700 and 1000 °C are shown in Table 2. The doping of NiFe_2_O_4_ with Zn^2+^, Mn^2+^, or Co^2+^ ions lead to a decrease of the M_S_ and M_R_ after annealing at 700 and 1000 °C. Above the single-domain critical size, the competition between the increasing magnetostatic energy and the domain-wall energy favors the domain-wall formation and the single-domain particle splits into multi-domain [9]. 

The low magnetization value of Co-NiFe_2_O_4_ is due to the incomplete crystallization and small-sized crystallites, which generate structural disorder on the nanoparticles surface. As the particles surface behaves as an inactive layer, its magnetization become negligible [5]. Some possible explanations for the variation of *Ms* in case of doped NiFe_2_O_4_ could be: (*i*) occupation of the octahedral sites by Zn^2+^ ions, (*ii*) random incomplete A–O–B linkages resulting in the replacement of non-magnetic ions by magnetic ions in the spinel, and (*iii*) the presence of non-collinear magnetic structures [16]. The magnetization caused by domain wall movement needs less energy than the domain rotation. The number of domain walls increases with increasing particle size. In case of Zn^2+^ doping, the wall movement contribution to magnetization is higher than that of the domain rotation [33]. Moreover, the presence of impurity phases with antiparallel magnetic ordering to the ferrite ordering reduces the *M_S_*. The doping with Co^2+^ ions having higher magnetic moment than Ni^2+^ ions result in a decrease of *M_S_*, as Ni^2+^ ion may occupy both the tetrahedral (A) and octahedral (B) sites [36]. 

By doping NiFe_2_O_4_, the *H_C_* decreases at 700 °C and increases at 1000 °C as a consequence of increased spin disorder in the surface layer and smaller particle size [1,32]. The *H_C_* value of 48 Oe of virgin NiFe_2_O_4_ decreases to 11 Oe in case of Co doping and annealing at 700 °C, most probably due to agglomerates’ formation which leads to the increase of average particles size above the critical single domain of NiFe_2_O_4_ particles and further leads to a multidomain magnetic structure [5]. Moreover, by annealing at low temperatures, the grain growth occurs, weakening the domain wall pinning effects at the grain boundary [5]. 

To calculate the magnetic anisotropy constant (*K*) of the samples, we assumed that the spinel ferrite particles have spherical shape. The *K* value of virgin NiFe_2_O_4_ is larger than that of doped NiFe_2_O_4_. The magnetic anisotropy of particles behaves as energy barrier and stops the switching of the magnetization’s direction to the easy axis [38,39]. At a certain temperature, the thermal activation overcomes the magnetic anisotropy energy barrier and the magnetization direction of the particles change, indicating a super-paramagnetic behavior [38,39]. A conceivable explanation could be the presence of a magnetically disordered surface layer, where a competition of exchange interactions between surface spins exists. Moreover, the magnetic disorder may originate in uneven magnetic interactions of the surface spins, arbitrarily oriented grains of different sizes and disordered vacancies [38,39].

## 4. Conclusions

The structural, morphological and magnetic properties of virgin and Zn-, Mn-, and Co-doped NiFe_2_O_4_ embedded in SiO_2_ matrix obtained through a modified sol-gel route and thermal treatment were investigated. The FT-IR spectra evidenced the formation of metallic precursors and of SiO_2_ matrix. The TG/DTA curves of samples dried at 40 °C indicated the formation and decomposition of metallic precursors to ferrites in single or two stages, with comparable mass losses. The XRD analysis revealed single-phase ferrites for virgin, Zn- and Co-doped NiFe_2_O_4_, and the presence of secondary crystalline phases derived from the SiO_2_ matrix (cristobalite, quartz, and Fe_2_SiO_4_) in case of Mn-doped NiFe_2_O_4_. XRD parameters were influenced not only by the crystallite size, lattice strain, and defects, but also by the annealing temperature and doping ions. The lattice constant and unit cell volume increase by doping with Mn^2+^ ion and decrease by doping with Zn^2+^ and Co^2+^ions. By contrast, X-ray and bulk densities, and porosity decrease by doping with Mn^2+^ and increase with doping Zn^2+^ and Co^2+^ ions. The NiFe_2_O_4_ particle size increases by doping with Mn^2+^ and decrease by doping with Zn^2+^ and Co^2+^ ions, respectively. The doping of NiFe_2_O_4_ with Zn^2+^, Mn^2+^ and Co^2+^ leads to a decrease of the saturation magnetization and remanent magnetization, whereas the coercivity decreases at 700 °C and increases at 1000 °C. The obtained magnetic transition metal dopped-Ni ferrite nanoparticles are possible candidates for various medical applications like controlled drug delivery, cancer therapy, biosensing, and magnetic resonance imaging.

## Figures and Tables

**Table 1:** Crystallite size (D), lattice constant (a), unit cell volume (V), bulk density (d_p_), X-ray density (d_XRD_), porosity (P), hopping length in tetrahedral sites (L_A_) and in octahedral sites (L_B_) and M/Ni/Fe molar ratio of virgin and doped NiFe_2_O_4_ (Zn-NiFe_2_O_4_, Mn-NiFe_2_O_4_, Co-NiFe_2_O_4_) NCs.

Temp (°C)	Sample	D (nm)	A (Å)	V (Å^3^)	d_p_ (g/cm^3^)	d_XRD_ (g/cm^3^)	P (%)	L_A_ (Å)	L_B_ (Å)	M/Ni/Fe Molar Ratio
400	NiFe_2_O_4_	11.5	8.4008	592.9	4.528	5.251	13.76	3.638	2.970	0/0.96/2.04
	Zn-NiFe_2_O_4_	4.4	8.3584	583.9	4.602	5.355	14.06	3.619	2.955	0.97/0.97/2.03
	Mn-NiFe_2_O_4_	16.5	8.4135	595.6	4.508	5.215	13.56	3.643	2.975	0.96/0.95/2.02
	Co-NiFe_2_O_4_	5.8	8.3462	581.4	4.582	5.356	14.45	3.614	2.951	0.97/0.96/2.03
700	NiFe_2_O_4_	18.2	8.4058	593.9	4.496	5.243	14.25	3.640	2.972	0/0.96/2.04
	Zn-NiFe_2_O_4_	6.7	8.3676	585.9	4.552	5.337	14.71	3.623	2.958	0.98/0.98/2.01
	Mn-NiFe_2_O_4_	24.6	8.4231	597.6	4.452	5.198	14.35	3.647	2.978	-
	Co-NiFe_2_O_4_	9.5	8.3923	591.1	4.488	5.267	14.79	3.634	2.967	0.99/0.98/2.01
1000	NiFe_2_O_4_	27.6	8.4182	596.6	4.411	5.219	15.48	3.645	2.976	0/0.99/2.00
	Zn-NiFe_2_O_4_	8.7	8.3824	589.9	4.478	5.301	15.52	3.630	2.964	0.99/1.00/2.00
	Mn-NiFe_2_O_4_	38.4	8.4295	599.0	4.401	5.186	15.13	3.650	2.980	-
	Co-NiFe_2_O_4_	20.2	8.4095	594.7	4.368	5.237	16.59	3.641	2.973	0.00/0.99/2.01

**Table 2:** Saturation magnetization (*M_S_*), remanent magnetization (*M_R_*), coercivity (*H_C_*), squareness (*S*), and anisotropy (*K*) of virgin and doped NiFe_2_O_4_ (Zn-NiFe_2_O_4_, Mn-NiFe_2_O_4_, Co-NiFe_2_O_4_) NCs.

Sample	*M_S_* (emu/g)		*M_R_* (emu/g)		*H_C_* (Oe)		*Sq*		*K*·10^3^ (erg/cm^3^)	
	700	1000	700	1000	700	1000	700	1000	700	1000
NiFe_2_O_4_	9.4	31.2	3.91	7.48	48	18	0.416	0.240	0.283	0.354
Zn-NiFe_2_O_4_	7.4	25.7	3.24	6.65	20	17	0.438	0.259	0.274	0.306
Mn-NiFe_2_O_4_	8.1	17.8	3.12	2.49	18	24	0.390	0.140	0.268	0.318
Co-NiFe_2_O_4_	3.8	16.2	0.26	3.84	11	32	0.068	0.237	0.026	0.326

## Data Availability

Not applicable.
